# Highly Pathogenic Avian Influenza H5N1 in Mainland China

**DOI:** 10.3390/ijerph120505026

**Published:** 2015-05-08

**Authors:** Xin-Lou Li, Kun Liu, Hong-Wu Yao, Ye Sun, Wan-Jun Chen, Ruo-Xi Sun, Sake J. de Vlas, Li-Qun Fang, Wu-Chun Cao

**Affiliations:** 1 State Key Laboratory of Pathogen and Biosecurity, Beijing Institute of Microbiology and Epidemiology, Beijing 100071, China; E-Mails: xinlou2010@163.com (X.-L.L.); liukun5959@163.com (K.L.); arenewu@163.com (H.-W.Y.); charlotte_fay@sina.com (Y.S.); wzmcwj@163.com (W.-J.C.); 1204081467@qq.com (R.-X.S.); 2 Department of Public Health, Erasmus Medical Center, University Medical Center Rotterdam, Rotterdam 999025, The Netherlands; E-Mail: s.devlas@erasmusmc.nl

**Keywords:** highly pathogenic avian influenza, H5N1, GIS, risk prediction, public health, China

## Abstract

Highly pathogenic avian influenza (HPAI) H5N1 has posed a significant threat to both humans and birds, and it has spanned large geographic areas and various ecological systems throughout Asia, Europe and Africa, but especially in mainland China. Great efforts in control and prevention of the disease, including universal vaccination campaigns in poultry and active serological and virological surveillance, have been undertaken in mainland China since the beginning of 2006. In this study, we aim to characterize the spatial and temporal patterns of HPAI H5N1, and identify influencing factors favoring the occurrence of HPAI H5N1 outbreaks in poultry in mainland China. Our study shows that HPAI H5N1 outbreaks took place sporadically after vaccination campaigns in poultry, and mostly occurred in the cold season. The positive tests in routine virological surveillance of HPAI H5N1 virus in chicken, duck, goose as well as environmental samples were mapped to display the potential risk distribution of the virus. Southern China had a higher positive rate than northern China, and positive samples were mostly detected from chickens in the north, while the majority were from duck in the south, and a negative correlation with monthly vaccination rates in domestic poultry was found (*R* = −0.19, *p* value = 0.005). Multivariate panel logistic regression identified vaccination rate, interaction between distance to the nearest city and national highway, interaction between distance to the nearest lake and wetland, and density of human population, as well as the autoregressive term in space and time as independent risk factors in the occurrence of HPAI H5N1 outbreaks, based on which a predicted risk map of the disease was derived. Our findings could provide new understanding of the distribution and transmission of HPAI H5N1 in mainland China and could be used to inform targeted surveillance and control efforts in both human and poultry populations to reduce the risk of future infections.

## 1. Introduction

Highly pathogenic avian influenza (HPAI) subtype H5N1 has posed significant threats to both humans and animals since the virus was initially detected in poultry on a farm of Scotland, UK [1] and it re-emerged in Southern China in the mid-1990s [2]. In 1997, HPAI H5N1 caused a serious outbreak at chicken farms and live bird markets in Hong Kong, where a total of 18 human infections were reported and six of them succumbed to the virus [2]. Since the re-emergence of the disease in 2003, the virus has spanned wide geographic regions and various ecological systems in countries of Asia, Africa and Europe with a high fatality rate in humans (approximately 60%) and in birds (100%) [3,4,5,6,7]. The direct impact involves birds’ deaths or culling to prevent the virus from spreading outside, and the loss of local and international trade of poultry and poultry products in the affected countries. The most troubling feature and unusual ability to cross the species barrier causing severe disease both in humans and other mammals have rightly received attention from the public, health experts, and political leaders as a potential pandemic threat [8].

Since the first outbreak in Guangxi in 2004, outbreaks associated with the HPAI H5N1 have been reported throughout the mainland China, which have caused more than 100 reported outbreak incidents in birds and more than 40 million poultry slaughtered for control and prevention of the disease [3,4]. In addition, human infections with avian influenza A (H7N9) and (H10N8)^9^, as well as outbreaks of H5N2 in poultry reported in mainland suggest the dynamic course and possibility of ongoing viruses’ reassortment. In the battle against HPAI H5N1, the government took great steps by implementing a universal vaccination campaign in poultry, which was a turning point in the control of the disease at the end of 2005. From the beginning of 2006, a national surveillance project has been established, which includes active routine virological and serological surveillance in birds, swine and related environments. The virological surveillance has been conducted to collect samples both at the provincial and national levels for the monthly detection of silent viral circulation. Samples were from six aspects including samples from chicken, duck, goose, wild birds, swine and other environmental samples. All samples are collected at the provincial level for detection of HPAI H5N1 virus by polymerase chain reaction (PCR). Serological surveillance has been conducted to collect samples from poultry at the provincial level for the estimation of vaccine efficacy. To answer the challenge of controlling HPAI H5N1 in mainland China where approximately 15 billion poultry feathers were produced annually, the important question is how to improve our understanding of the spatial and temporal distribution patterns and influencing factors favoring the continuing reoccurrence of the HPAI H5N1in mainland China.

It was evidenced that HPAI H5N1 outbreaks have a presence throughout a variety of ecological systems with considerable economic, agricultural and environmental differences. In previous work, various approaches have been used and demonstrated that the risk of HPAI H5N1 outbreaks in poultry was associated with national highways, interactions between lakes and wetlands, and precipitation in mainland China [10], and the number of free-ranging duck in Thailand [11,12] and the local abundance of both ducks and geese in Vietnam [13], in addition to other risk factors such as number of chickens, density of human population in southeast Asia [14], and topographical features [14], as well as the highway network in Nigeria [15]. It has also been found that HPAI H5N1 outbreaks in domestic poultry were associated with chicken density, human population density, and elevation, and the occurrence of HPAI H5N1 in surveillance was associated with density of domestic waterfowl, density of human population and the percentage coverage of water bodies in China [16]. It also has been observed HPAI H5N1 continues to circulate with a seasonal pattern [17,18], and the earlier occurrence of HPAI H5N1 outbreaks in domestic poultry was proved to be significantly associated with higher temperature [19]. Some studies have also demonstrated that migratory birds were considered as the natural reservoir and play a role in spreading of HPAI H5N1 [20,21,22,23]. However, those analyses were mainly based on cross-sectional data. While the HPAI H5N1 persists endemically and shows occasional resurgence in mainland China, and the virus underwent a series of evolutionary changes, and the ecological environment as well as disease management during the epidemics are changing as a result, which prompts the need for risk factor analysis to take account of these changes.

In this study, we aim to characterize explicitly the temporal and spatial pattern of HPAI H5N1 in mainland China from 2004 to 2012, and analyze the interrelationship between HPAI H5N1 and environmental and socio-economic indicators so as to provide essential information for developing effective and appropriate countermeasures against HPAI.

## 2. Materials and Methods

### 2.1. Data on HPAI H5N1

Data on HPAI H5N1 outbreaks in poultry and wild birds were officially reported by the Ministry of Agriculture of China (MoA). According to the guideline “Technical requirement for prevention and control of highly pathogenic avian influenza” issued by the MoA, an outbreak of HPAI H5N1 in poultry or wild birds is defined as a positive test for avian influenza A (H5N1) virus for any sick poultry or birds by laboratory based on virological methods in a location (usually a poultry farm or a village) [24]. The information collected included bird type, date of symptom onset, the latitude and longitude of the outbreak and number of domestic poultry deaths. Also, data on monthly virological and serological routine surveillances for HPAI H5N1 were obtained from the national surveillance project by the MoA, which includes monthly vaccination rates, number of samples from chicken, duck, wild birds, swine and related environment samples for virological detection and positive results, for each Chinese province from 2006 to 2012 [25]. We also collected the data on laboratory-confirmed human infections from updates published by World Health Organization (WHO) in this study [26].

### 2.2. Ecologic Variables

To identify influencing factors favoring the occurrence of the HPAI H5N1outbreaks in poultry in mainland China, agro-ecological, environmental and climate variables were collected based on previous studies [10,11,13,14,15,16,17,18,19,27,28,29,30], including transportation routes, location of main cities, distribution of water bodies (areas of more than 10 km^2^), distribution of wetlands, density of human population, poultry density, normalized difference vegetation index (NDVI), land cover, mean elevation and climate variables. Transportation routes and main cities were obtained as in our previous studies [10]. Information on the water bodies and wetlands was obtained from the State Key Lab of Remote Sensing Science as previous described [10]. Data on density of human population in 2010 were obtained from the Data Sharing Infrastructure of Earth System Science (http://www.geodata.cn/). Poultry density in 2009 was obtained from the Food and Agriculture Organization of the United Nations [31]. NDVI was generated from “Free Vegetation Products” (http://free.vgt.vito.be) in digital number values, and monthly maximum values were calculated. To extract the percentages of coverage of croplands, grassland, shrub, forests and buildup, the land cover data from 2005 were collected from the Data Sharing Infrastructure of Earth System Science (http://www.geodata.cn). Elevation rasters with a spatial resolution of 1 × 1 km^2^ were obtained from the Global Digital Elevation Data Products (http://www.gscloud.cn). Climate variables, including monthly temperature and monthly precipitation from more than 800 weather stations were obtained (www.cdc.cma.gov.cn) and extrapolated by the kriging technique in ArcGIS 9.3 (ESRI Inc., Redlands, CA, USA).

### 2.3. Spatiotemporal Analysis

The epidemic bar charts of monthly officially reported outbreaks in domestic poultry and wild birds, monthly reported human infections and positive rate in virological surveillance were created to explore the temporal pattern of HPAI H5N1 epidemic in mainland China from 2004 to 2012. To explore the spatial distribution pattern of HPAI H5N1, the spatial distribution of outbreaks in wild birds, reported human infections and number of deaths in domestic poultry during the HPAI outbreaks were geo-referenced and linked to the digital map of China (www.geodata.cn) according to their symptom onset locations using GIS technologies as we have done in previous studies [10,27]. The poultry density was overlapped as the background map. The number of positive rates from different species in virological surveillance was also mapped at the province level, and vaccination rates were overlapped as the background map. In order to understand the seasonal distribution of both reported outbreaks in domestic poultry and positive samples in virological surveillance, we divided the different seasons of outbreaks and positive rate according to their onset date and illustrated the seasonal distribution for positive rate at the province level and seasonal distribution of HPAI H5N1 outbreaks by using the symptom onset locations.

### 2.4. Analysis of Potential Factors Associated with the HPAI H5N1 Outbreak in Poultry

In order to identify the potential factors with dynamic presence of HPAI H5N1 outbreaks in poultry in mainland China, case-control design and panel logistic regression were developed which benefit from previous studies with a spatial resolution of 10 km × 10 km and a temporal resolution of a year [10,16,27]. Reported outbreaks in poultry onset symptoms data from 2004 to 2008 were involved in the analysis to build the regression model. Each outbreak was taken as a “case”, and 5-fold controls for each case were randomly selected from throughout the mainland China based on these conditions [10,16,27]: (1) being at a minimum distance >10 km of any “case”; (2) being in a location where there were more than one head of domestic poultry; (3) no HPAI H5N1 outbreaks had been reported during the period. All cases and controls were taken as panel, and a year was taken as the time interval. Potential factors (vaccination rate, transportation routes, water bodies, wetland, density of human population, density of poultry, climate variables, land cover and autocorrelation) involving 25 variables were included in the study. Yearly data of all involved variables were collected from 2004 to 2008. Distance to the nearest transportation routes including highway, railway and freeway, were calculated by proximity tools in ArcGIS 9.3 as the minimal distance from each case and control grid center to its nearest transportation routes. Distance to the nearest city was calculated by using the grid center of “cases and controls” to the nearest city center. Distance to the nearest water bodies including lake, water reservoir, river and wetland, were measured using the proximity function from each case and control grid to the nearest water body edge. Furthermore, zonal statistical calculation was used to calculate variables with 8 km mean buffer zone [10,16]. NDVI were calculated each year. Density of human population, density of poultry and percentages coverage of croplands, grassland, shrub, forests, buildup and mean elevation for each case and control grid were zonal statistic by spatial analyst tools in ArcGIS 9.3. To evaluate the probability of presence/absence of outbreak in each 10 square km over a year in this study, we considered the probable seasonal effect of meteorological factors on HPAI H5N1 outbreaks and climate variables including mean temperature in summer and in winter, precipitation in spring and summer, and precipitation in autumn and winter. We also included an autocorrelation term which accounts for spatial and temporal dependency in response for spatial-temporal distribution of infectious diseases which have been demonstrated in other studies [32,33,34]. We assumed that the previous outbreaks have effects on the followed outbreaks in both spatial and temporal in spread and transmission of avian influenza. In this study, the autocorrelation term was developed to examine the effects from previous outbreaks to coming outbreaks. The autocorrelation term was calculated using the mean distance from each case and control to the nearest previous three outbreaks which was divided 10 and took a reciprocal transform. The calculation was performed by Hawth’s tools in ArcGIS 9.3. We combined all the prepared data into the panel dataset.

In performing the panel logistic regression, univariate analysis was firstly performed to examine the effect of each variable separately. ORs (odds ratio) of the variables involving distance to nearest transportation routes, distance to the nearest city, distance to the nearest water body and distance to the nearest wetland were calculated for a ten-kilometers difference, and 10% difference for vaccination rate, 1000-persons per km^2^ for density of human population, 100-head per km^2^ for density of poultry, one-centigrade for temperature, 100-millimeter difference for precipitation, 10% change for each type of land cover and 100-meter for mean elevation were respectively used in the analysis. Multivariate analysis was then performed using variables with *p*-value < 0.1 from the univariate analyses as covariates. The possible interactions between each covariate were also included in the analysis as we done in previous study [10]. Continuous variables were also presented categorical results to allow inspection of the data and determine whether or not the assumption regarding continuous variables was justified. If the significant non-linear associations between HPAI H5N1 outbreaks and the variables were found, we included them combined with a polynomial structure in the analysis. Models were also optimized by comparing the −2 log likelihood and goodness of fit when continuous or categorical variables that having a non-linear association with the outbreaks of HPAI H5N1 were respectively added or removed from the models, but no finer models were found by including categorical structure of them.

A predictive map based on the final model of the panel logistic regression was created and classified each grid as the highest risk, higher risk, medium risk and low risk according different level for predicted probability of HPAI H5N1 presence in poultry across the mainland China. Statistical analyses were performed in STATA software (Stata Corp LP, College Station, TX, USA). Panel logistic regression was performed, and odds ratios (ORs), their 95% confidence intervals (CIs) and *p*-values were estimated using maximum likelihood methods. The predictive probability map was plotted in ArcGIS 9.3.

## 3. Results

**Figure 1:**
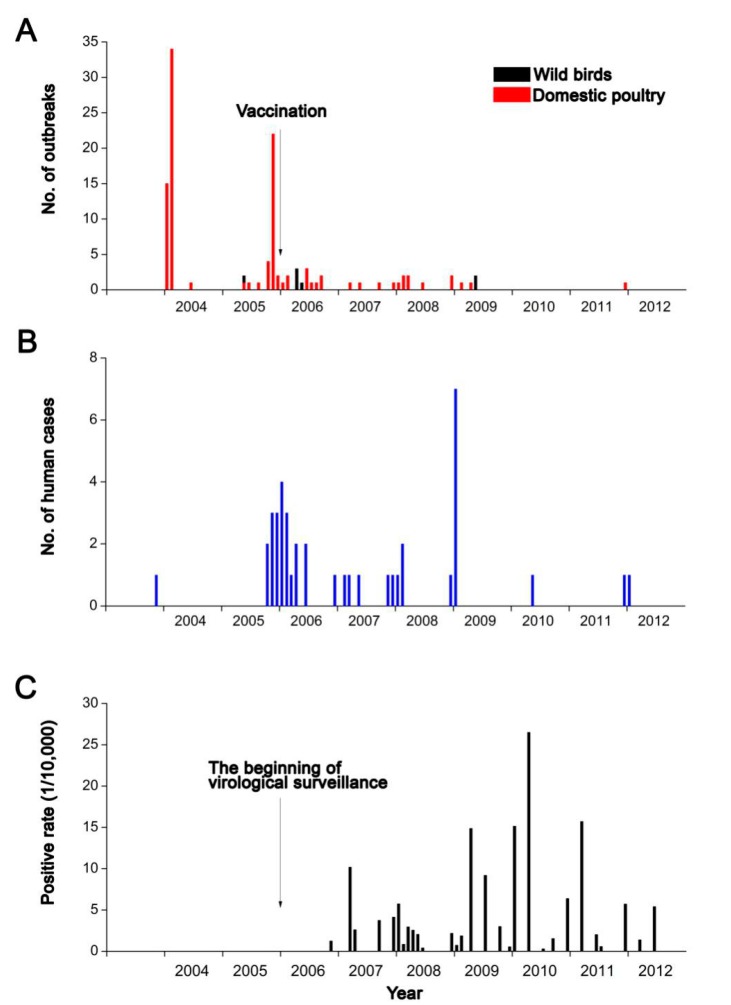
Temporal distribution of HPAI H5N1 in mainland China. (**A**) Outbreaks in domestic poultry and wild birds. The red and black bar charts indicate the number of outbreaks in domestic poultry and in wild birds, respectively. The black arrow indicates the beginning of vaccination campaign in domestic poultry in mainland China; (**B**) Number of human cases with HAPI H5N1 infection; (**C**) Positive rate in virological surveillance. The black bar charts indicate the positive rate in virological surveillance, and the black arrow indicates the beginning of the routine virological surveillance in mainland China.

Since the emergence of HPAI H5N1 outbreaks in poultry in mainland China in January 2004, a total of 106 and seven outbreaks, respectively, in domestic poultry and wild birds were reported in mainland China as of the end of 2012, which were located in 140 counties of 24 provinces, causing the death of about 400,000 birds and the destruction of about 30.6 million domestic birds. In addition, 41 laboratory-confirmed human cases caused by the virus were reported during the study period. After two epidemic peaks of HPAI H5N1 in domestic poultry in January–February 2004 and November 2005, respectively, the HPAI H5N1 epidemic in domestic poultry decreased notably but still occurred sporadically since the end of 2006, and all seven presences of HPAI H5N1 in wild birds occurred in April or May (Figure 1A). Human infections were still seasonal and the majority of them were reported in the cold season, being similar to the outbreaks in domestic poultry (Figure 1B). The bar chart of positive rate in virological surveillance shows a different temporal pattern with outbreaks in domestic poultry and human infections and indicates that HPAI H5N1 virus could be still detected in birds or related environments, although there were few outbreaks in domestic poultry and human cases reported after 2009 (Figure 1C).

In the national surveillance project, a total of 501 positive samples were detected from 2,905,857 samples, in which duck and chicken accounted for 51.90% (260/501) and 39.72% (199/501), respectively. Another 19, 20 and three positive samples were detected from goose, wild birds, and related environments, respectively.

In addition, no positive samples were detected in swine. The outbreaks of HPAI H5N1 in domestic poultry mainly took place in the cold season, with four months (November, December, January and February), accounting for 79.2% (84/106) of all outbreaks, while a few appeared in warm months, with July, August, and September accounting for only 5.7% (6/106) of all outbreaks. The detailed information on the number of outbreaks involving domestic poultry, and the number of deaths and destroyed poultry for each month is summarized in Table 1.

**Figure 2:**
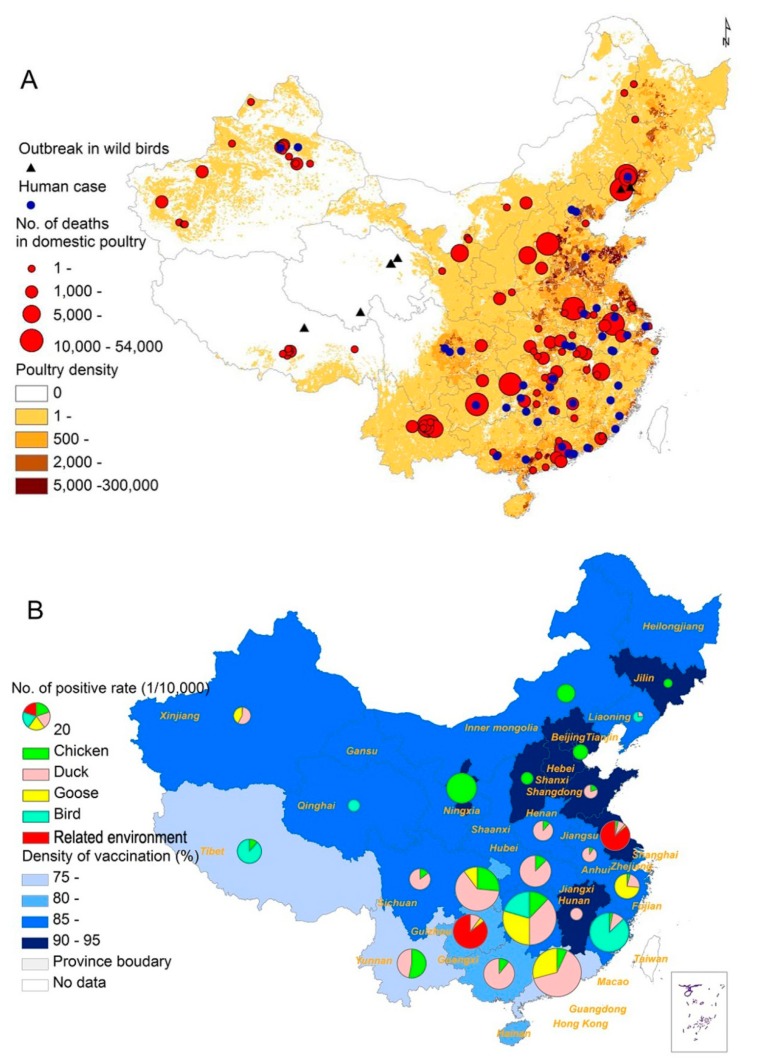
Spatial distribution of HPAI H5N1in mainland China, 2004–2012; (**A**) Spatial distribution of HPAI H5N1 outbreaks in wild bird, human cases and number of deaths in domestic poultry. Black triangle indicates the location of HPAI H5N1 outbreak in wild birds, blue dot indicates the location of human case, and red dot indicates the location and number of deaths of domestic poultry. Density of poultry is indicated by color gradient; (**B**) Positive rate in surveillance from difference species. Light apple, rose quartz, autunite yellow, beryl green and red pies indicate the positive rate in chicken, duck, goose, wild birds and related environment, and density of vaccination was indicated by blue color.

The spatial distribution map of HPAI H5N1 in birds and human cases shows that the disease has spanned a large geographic space and caused a number of deaths in domestic poultry, and human cases (43.9%) were partly located in these prefectures with HPAI H5N1 outbreaks in birds (Figure 2A). The virological surveillance data showed that southern China had a higher positive rate than northern China, and positive samples were mostly detected from chicken in the north, while in the south the majorities were from duck (Figure 2B). Positive results were also detected from related environmental samples in Jiangsu and Guizhou provinces.

**Table 1:** Summary of outbreaks of HPAI H5N1 in domestic poultry for each month.

Month	Outbreaks (%)	Species of Domestic Poultry	Deaths (Feature)	Destroyed (Feature)
January	17 (16.04%)	Chicken, duck, and goose	51,638	1,295,494
February	39 (36.79%)	Chicken & goose	131,029	4,131,160
March	3 (2.83%)	Chicken	1062	9558
April	1 (0.94%)	Chicken	1500	1679
May	2 (1.89%)	Duck & goose	11,632	66,331
June	6 (5.66%)	Chicken, duck, and goose	14,799	2,393,358
July	1 (0.94%)	Chicken	3045	356,976
August	2 (1.89%)	Chicken	1938	295,805
September	3 (2.83%)	Chicken & duck	11,815	234,920
October	4 (3.77%)	Chicken, duck, and goose	12,655	6,138,323
November	22 (20.75%)	Chicken, duck, and goose	133,777	15,799,404
December	6 (5.66%)	Chicken, duck, and goose	8873	194,930

A total of 31,525,947 samples were collected and tested in the serological surveillance, and the protective samples accounted for 89.6% (28,259,278/31,525,947). In general, the vaccination rates varied across provinces (Figure 2B), and a negative correlation between monthly positive rates in routine virological surveillance of HPAI H5N1 virus and monthly vaccination rates in domestic poultry was found by Spearman correlation analysis (*R* = −0.19, *p* value = 0.005).

**Figure 3:**
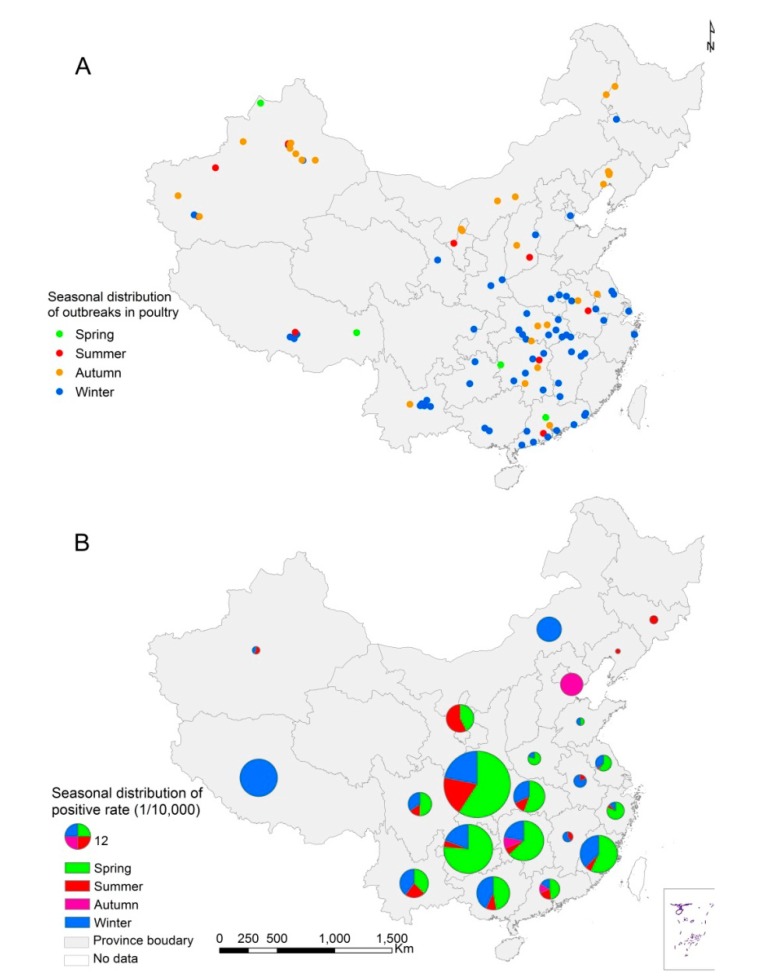
Seasonal distribution of HPAI H5N1 outbreaks and positive samples at province-level; (**A**) Seasonal distribution of HPAI H5N1 outbreaks in domestic poultry, 2004–2012; (**B**) Seasonal distribution of positive rate in surveillance, 2006–2012.

Figure 3 shows the seasonal distribution of outbreaks in birds and positive results in the virological surveillance in mainland China. In the southern China, outbreaks in domestic poultry were mainly reported in winter and spring, while majority of the outbreaks in northern China occurred in autumn (Figure 3A). In addition, positive samples in the virological surveillance were detected mainly in spring and winter seasons (Figure 3B).

Panel logistic regression analysis shows that the HPAI H5N1 outbreaks in domestic poultry were significantly associated with vaccination rate, distance to the nearest highway, railway, main city, lake and wetland, as well as density of human population, density of poultry, mean temperature in summer, mean temperature in winter, precipitation in spring and summer, land cover (percentage coverage of cropland, grassland, shrub and buildup), mean elevation and the autoregressive term in the univariate analysis (Table 2).

In the multivariate analysis, five variables including vaccination rate, interaction between distance to the nearest city and national highway, interaction between distance to the nearest lake and wetland, and density of human population, as well as the autoregressive term were found to be independent risk factors in the occurrence of HPAI H5N1 outbreaks (Table 2). The ORs for the vaccination rate, interaction between distance to the nearest city and national highway, and interaction between distance to the nearest lake and wetland were 0.79 (*p* value < 0.001), 0.96 (*p* value = 0.002) and 0.87 (*p* value < 0.001) respectively. HPAI H5N1 outbreak was also influenced by density of human population (OR = 1.44, *p* < 0.001) and the autoregressive term (OR = 1.40, *p* value < 0.001).

**Table 2:** The association between HPAI H5N1 outbreaks and potential factors by panel logistic regression analysis.

Variables ( Unit ) ^a^	No. of Outbreaks (95% CI)	Univariate Analysis			Multivariate Analysis	
		OR (95% CI)	*p* Value		OR (95% CI)	*p* Value
Vaccination rate (categorical, %)						
<70	85 (68, 102)					
70–	13 (6, 20)					
>90	5 (1, 9)					
Vaccination rate (continuous, 10 %)		0.80 (0.76, 0.85)	<0.001		0.79 (0.74, 0.84)	<0.001
Distance to the nearest transportation routes						
National highway (categorical, 10 km)						
<1	57 (43, 71)					
1–	36 (24, 48)					
>3	10 (2, 12)					
National highway (continuous, 10 km)		0.66 (0.57, 0.77)	<0.001			
Railway (categorical, 10 km)						
<3	87 (69, 105)					
3–	10 (4, 16)					
>10	6 (1, 10)					
Railway (continuous, 10 km)		0.91 (0.87, 0.96)	<0.001			
Freeway (categorical, 10 km)						
<3	83 (66, 100)					
3–	11(5, 17)					
>50	9 (3, 15)					
Freeway (continuous, 10 km)		0.99 (0.98, 1.00)	0.217			
Distance to the nearest city (categorical, 10 km)						
<5	72 (56, 88)					
5–	24 (14, 34)					
>10	7 (2, 12)					
Distance to the nearest city (continuous, 10 km)		0.78 (0.73, 0.84)	<0.001			
Interaction between distance to the nearest city and national highway (continuous, 10 km * 10 km)		0.93 (0.91, 0.96)	<0.001		0.96 (0.94, 0.99)	0.002
Distance to the nearest water body						
Lake (categorical, 1 km)						
<30	96 (77, 115)					
30–	5 (1, 9)					
>50	2 (0, 5)					
Lake (continuous, 10 km)		0.60 (0.49, 0.72)	<0.001			
Water reservoir (categorical, 10 km)						
<10	83 (66, 100)					
10–	12 (5, 19)					
>30	8 (3, 13)					
Water reservoir (continuous, 10 km)		0.95 (0.91, 1.00)	0.031			
River (categorical, 10 km)						
<3	64 (49, 79)					
3–	29 (19, 39)					
>10	10 (4, 16)					
River (continuous, 10 km)		0.99 (0.98, 1.01)	0.491			
Distance to the nearest wetland (categorical, 10 km)						
<2	88 (70, 106)					
2–	13 (6, 20)					
>5	2 (0, 5)					
Distance to the nearest wetland (continuous, 10 km)		0.60 (0.48, 0.75)	<0.001			
Interaction between distance to the nearest Lake and wetland (10 km * 10 km)		0.76 (0.67, 0.85)	<0.001		0.87 (0.78, 0.96)	<0.001
Density of human population (categorical, 1000 persons per km^2^)						
<0.1	16(8, 24)					
0.1–	32(21, 43)					
>0.4	55(41, 69)					
Density of human population (continuous, 1000 persons per km^2^)		1.66 (1.42, 1.95)	<0.001		1.44 (1.19, 1.73)	<0.001
Density of poultry (categorical, 100 poultry per km^2^)						
<1	28(18,38)					
1–	26(16,35)					
>5	49(36,62)					
Density of poultry (continuous, 100 poultry per km^2^)		1.02 (1.01, 1.03)	<0.001			
Quadratic density of poultry (continuous)		1.00 (1.00, 1.00)	0.082			
Climate						
Mean temperature in summer (categorical, 1 centigrade)						
<20	11(5, 17)					
20–	33(22, 44)					
>25	59(44, 74)					
Mean temperature in summer (continuous, 1 centigrade)		1.11 (1.04, 1.17)	0.001			
Mean temperature in winter (categorical, 1 centigrade)						
<0	7 (2, 12)					
0–	25 (15,35)					
>10	71 (55, 87)					
Mean temperature in winter(continuous, 1 centigrade)		1.04 (1.02, 1.07)	<0.001			
Precipitation in spring and summer(categorical, 100mm)						
<1	7 (2,12)					
1–	29 (19, 39)					
>5	67 (51,83)					
Precipitation in spring and summer(continuous,100mm)		1.07 (1.02, 1.12)	0.004			
Precipitation in autumn and winter (categorical, 100mm)						
<0.5	13 (6, 20)					
0.5–	28 (18, 38)					
>2	62 (47,77)					
Precipitation in autumn and winter (continuous,100mm)		1.12 (0.99, 1.27)	0.064			
NDVI (categorical)						
<100	28 (18, 38)					
100–	42 (30, 54)					
>150	33 (21, 44)					
NDVI (continuous)		1.00 (1.00, 1.01)	0.556			
Quadratic NDVI (continuous)		1.00 (1.00, 1.00)	0.808			
Land use (10 %)						
Percentage coverage of cropland (categorical, 10%)						
<1	15 (7, 21)					
1–	34 (23, 45)					
>5	55 (41, 69)					
Percentage coverage of cropland (continuous, 10%)		1.18 (1.11, 1.25)	<0.001			
Percentage coverage of grassland (categorical, 10%)						
<2	59 (44, 74)					
2–	31 (20, 42)					
>5	13 (6, 20)					
Percentage coverage of grassland (continuous, 10%)		0.91 (0.84, 0.99)	0.022			
Percentage coverage of shrub (categorical, 10%)						
<0.05	56 (42, 70)					
0.05–	34 (27, 45)					
>0.1	13 (6, 20)					
Percentage coverage of shrub (continuous, 10%)		0.66 (0.52, 0.85)	0.001			
Percentage coverage of forest (categorical, 10%)						
<0.05	59 (44, 74)					
0.05–	25 (15, 35)					
>0.5	19 (11, 27)					
Percentage coverage of forest (continuous, 10%)		0.88 (0.76, 1.03)	0.104			
Percentage coverage of buildup (categorical, 10%)						
<0.05	36 (24, 48)					
0.05–	35 (24, 46)					
>0.5	32 (21, 42)					
Percentage coverage of buildup (continuous, 10%)		1.52 (1.31, 1.76)	<0.001			
Mean elevation (categorical , 100 m)						
<5	66 (50, 82)					
5–	29 (19, 39)					
>20	8 (2, 14)					
Mean elevation (continuous , 100 m)		0.96 (0.94, 0.99)	0.002			
Autoregressive term (continuous)		1.50 (1.39, 1.63)	<0.001		1.40 (1.28, 1.54)	<0.001

**^a^** For all continuous variables, categorical results are also reported to allow inspection of the data and assessment of whether or not the assumption regarding continuous variables was justified.

**Figure 4:**
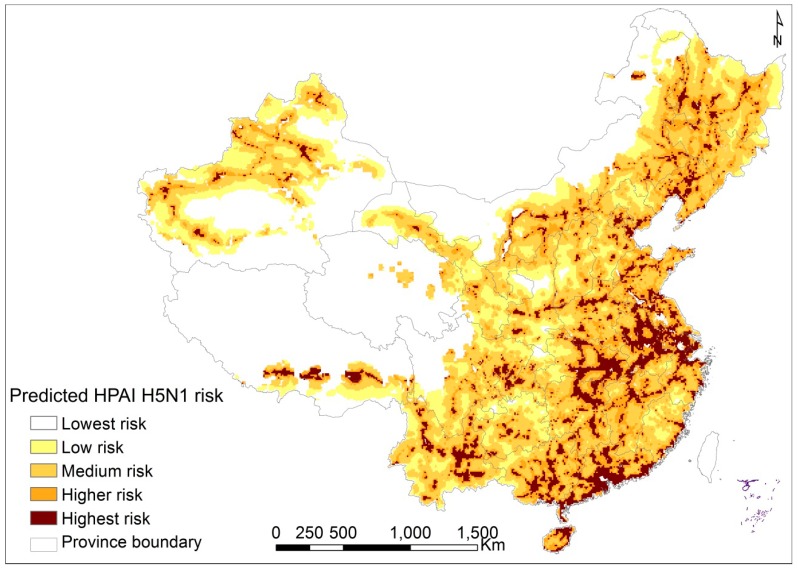
Predictive risk of HPAI H5N1 outbreaks in domestic poultry in mainland China; The predictive risk map shows increased risk of HPAI H5N1 outbreaks in domestic poultry with color gradient.

The predictive map shows that the southern, east-central China and some areas in northeastern China had higher risk of HPAI H5N1 outbreaks in domestic poultry (Figure 4). We note a marked difference for the outbreak data compared with previous works [10,16].

## 4. Discussion

The emergence of HPAI H5N1 in mainland China has caused heavy losses and posed significant threats to both humans and poultry. In this study, we provide an overview of the temporal and spatial distribution of HPAI H5N1 in mainland China, and identify the factors favoring the occurrences of HPAI H5N1 outbreaks in domestic poultry by using a dynamic regression model, and a predictive risk map was created to display the risk distribution of HPAI H5N1 outbreaks in domestic poultry.

Since the first outbreak official reported in 2004 in Guangxi (China), HPAI H5N1 has caused great concern. Control measurement was taken and large-scale poultry vaccination against HPAI H5N1 was introduced at the end of 2005, as well as a national surveillance project at the beginning of 2006, which have impacted the disease epidemic dramatically. It is remarkable that vaccination and early detection in virological surveillance worked and almost no outbreaks were reported in domestic poultry after 2009 (Figure 1A). While the positive results in the routine surveillance show the HPAI H5N1virus has still been in existence in domestic poultry, especially in southern China, which indicates that the disease persists endemically, attention needs to be paid to the occasional resurgence of the disease.

After serious outbreaks, studies aiming at identifying the agro-ecological, environmental, and meteorological factors favoring the occurrence of HPAI H5N1 outbreaks have been undertaken in mainland China [10,16,29], and attempts have been made to created risk maps of HPAI H5N1 outbreaks [10,16]. However, taking account the changes in agro-ecological, environmental, and climate factors, as well as control measurement, this is more necessary. The study benefits from previous works and involves several improvements [10,16,29].

Our studies demonstrated that the HPAI H5N1 outbreaks in poultry and human infections spanned throughout mainland China. In addition, positive virological surveillance samples were detected from a large geographic range throughout the mainland and varied across species, including samples from domestic poultry, wild birds, as well as environmental samples. A higher positive rate in duck in southern China was observed, which has demonstrated that HPAI H5N1 virus tends to display low pathogenicity in waterfowl and can maintains virus circulation, indicating that it’s difficult to eliminate thoroughly the spread of the virus. Swine was proved to support circulation of influenza viruses which should be stressed, although no positive samples were detected in swine during surveillance from 2006 to 2012. Many outbreaks were observed in winter throughout southern China [18], indicating that climate plays an important role and has an impact on the spatiotemporal distribution of HPAI H5N1 [34]. Our panel logistic regression demonstrated that the HPAI H5N1 outbreaks in domestic poultry were significantly associated with vaccination rate, interaction between distance to the nearest city and national highways, interaction between distance to nearby lakes and wetlands, and density of human population, as well as the autoregressive term. The association between presence of HPAI H5N1 outbreaks and vaccination rate was negative, which indicated the nationwide massive vaccination campaign for the prevention of HPAI H5N1 in domestic poultry has worked. The transportation routes play roles in preceding the presence of HPAI H5N1 as has been demonstrated in previous works in China and Nigeria [10,15], with national highways being taken as an indicator of the intensity of local and long-distance trade of live poultry and poultry products across the country, which indicated that trade and movement of poultry can help the long-distance spread of HPAI H5N1 [10]. Major cities play a role in the spread of HPAI H5N1due to the frequent poultry trade around them as well as a large density of human population, which can result in frequently increased virus circulation and transmission around them and their surroundings [29]. Water bodies and wetlands have been regarded as an integral part of the waterfowl habitat, e.g., domestic ducks and goose. Lakes, reservoirs and rivers can provide a habitat for wild birds as well as domestic waterfowl [10,16,30]. Wetland can also provide food and habitat for migratory birds and some poultry species. Infected migratory birds have been reported to spread the virus for long distances, and the fact that they search for food and shelter in wetlands and other water bodies may increase the frequent contact between wild birds and domestic poultry [10,16]. Some domestic poultry species can become infected after drinking infected water. Density of human population can be a predictor of higher probability of detection and reporting, as well as trade-related factors. Populated places also correspond to poultry marketing activities and intensive poultry trade and demand for more poultry products, which can increase the risk of transmission and spread [30]. The autoregressive term accounts for the spatial dependency which has been demonstrated by Gilbert in Southeast Asia [14]; our studies demonstrated autoregressive term was positive associated with HPAI H5N1 in mainland China, indicating that viral circulation from host and environment once established can persist in their surroundings which also supports our assumption that the previous HPAI H5N1 outbreaks have impacted subsequent outbreaks both spatially and temporally.

It has been demonstrated that wild bird migration can act as the natural reservoir of avian influenza viruses and can carry the viruses across large geographic regions without being detected during their migration [20,21,22,23]. In our previous work [10], we could not find the association between the HPAI H5N1 outbreaks and bird migration routes due to lack of detailed data about bird migration routes in mainland China. The panel logistic regression result in the study demonstrates that presence of HPAI H5N1 is not significantly associated with the density of poultry in multivariate analysis, which is due to the fact that industrialized farms with high poultry densities also typically have relatively high levels of bio-security, hygiene and disease prevention practices [10,13,27]. Seasonality of HPAI H5N1 outbreaks in domestic poultry and viral isolation has been documented [35]. Our study demonstrates that presence of HPAI H5N1 is not significantly associated with either temperature or precipitation; however, climate can alter bird migration patterns, as well as the virus transmission cycle [36,37]. More accurate variables which can describe the effect of climate change will be a study focus in the future. We also reported no significant association between vaccination rate and scales of HPAI H5N1 outbreaks as correlated the number of deaths in domestic poultry and vaccination rates. The scale of outbreaks should be linked with the number of domestic poultry in the location and the time duration from onset to detection of the outbreak, as well as the time interval between control measurements, which should be taken into consideration in any future analysis. Some studies have demonstrated that clade type and control measurement have impact on the transmissibility of HPAI H5N1 virus during different epidemic waves [20,22], which also should be taken into account in future analysis. Some methods including phylogenetic and spatial phylogenetic can also improve our understanding of the spread of avian influenza viruses [20,38]. According to the predictive map established in our analysis, areas at highest risk were identified and concentrated in part of Shanghai, Jiangsu, and Anhui in south-eastern China and Guangdong, and Guangxi in southern China. Particular attention should be paid to those places.

## 5. Conclusions

This study characterized the temporal and spatial patterns of HPAI H5N1 outbreaks, human infections and positive virological surveillance results in mainland China. Five influencing factors contributing to the presence of HPAI H5N1 in domestic poultry were identified in this study, which include vaccination rate, interaction between distance to the nearest city and national highways, interaction between distance to the nearest lake and wetland, and density of human population, as well as the autoregressive term in space and time. The predictive map shows the spatial risk distribution of HPAI H5N1 occurrence in domestic poultry in mainland China. Our findings could be helpful for understanding of the distribution and transmission of HPAI H5N1 and could be used to inform targeted surveillance and control efforts in both human and poultry populations to reduce the risk of future infections.
